# A New Approach in PLS/TPS Compatibilization Using Garlic Oil: Effect on Morphological and Antioxidant Properties

**DOI:** 10.3390/antiox13121589

**Published:** 2024-12-23

**Authors:** Maria Cidália R. Castro, Pedro Veiga Rodrigues, Vasco Cruz, Ana Vera Machado

**Affiliations:** Department of Polymer Engineering, Institute for Polymers and Composites (IPC), University of Minho, 4804-533 Guimarães, Portugal; pedro.rodrigues@dep.uminho.pt (P.V.R.); vasco.cruz@dep.uminho.pt (V.C.); avm@dep.uminho.pt (A.V.M.)

**Keywords:** biodegradable polymers, compatibilization, garlic oil, antioxidant, food packaging

## Abstract

The packaging industry has made efforts to reduce food waste and improve the resilience of food systems worldwide. Active food packaging, which incorporates active agents, represents a dynamic area where industry and academia have developed new strategies to produce innovative and sustainable packaging solutions that are more compatible with conventional options. Due to health and environmental concerns, industries have sought alternatives to petroleum-based materials and have found biopolymers to be a viable option because of their biodegradable and safe nature. The combination of PLA/TPS has emerged as an effective system for packaging film; however, they are thermodynamically immiscible. This work highlights the development of a starch-based compatibilizer to connect the PLA and TPS phases by functionalizing maize starch with glycidyl methacrylate, glycerol, or garlic oil. Garlic oil was chosen for its plasticizing ability and antioxidant properties. The films produced exhibited excellent compatibility, with enhanced interfacial adhesion between PLA and TPS components. The introduction of compatibilizers also increased the systems’ crystallinity and improved their mechanical properties. The wettability of the films significantly increased with higher garlic oil content, along with enhanced antioxidant properties. These advancements will enable the production of a compatible PLA/TPS system with improved properties for application in the packaging industry.

## 1. Introduction

Food waste has significant environmental, social, and economic repercussions. In 2024, 8% to 10% of global greenhouse gas emissions were linked to unconsumed food, including waste before consumption due to expiration dates [1]. A significant amount of packaged food is wasted due to various environmental factors, including moisture, oxidation, UV-Vis irradiation, and microbial contamination. Therefore, prioritizing investment in methods to minimize food waste is crucial. One effective approach is to improve food packaging to better preserve and enhance the quality, safety, and shelf-life of food [2]. Furthermore, the fast pace of everyday life increasingly demands high-quality and safe food options to save consumers time. Over the years, the food packaging industry has worked to meet these demands by developing new strategies and technologies. Active packaging is an effective technology used in food packaging to address these issues. It interacts with the product to extend its shelf-life and ensure quality [3,4]. Active packaging incorporates selected agents, typically with biological activity, that release substances into or from the package or the surrounding environment. Among these agents, those that prevent food oxidation or inhibit microbial growth have received particular attention, as they help prevent the deterioration of food products [5,6,7,8]. Natural compounds with antioxidant properties, derived from plants, animals, or soils, are widely used in the food industry for food preservation and packaging [6,7]. Garlic, a well-known natural product in culinary and medicinal fields, contains organosulfur and phenolic compounds responsible for its health benefits and antioxidant properties [9,10]. Garlic extract is already widely used to produce edible films in the food industry [11,12,13,14,15].

In recent years, the food packaging industry has undergone a significant shift due to societal demands and ecological concerns. This change has prompted researchers to develop sustainable and biodegradable packaging materials, particularly for single-use items, as alternatives to petroleum-based polymers [16]. Biodegradable polymers, such as polylactic acid (PLA) and thermoplastic starch (TPS), have gained significant attention from both academia and industry due to their biodegradability, eco-friendliness, nontoxicity, and biocompatibility. While PLA shares mechanical properties with conventional polymers, TPS is more cost-effective, making it an attractive commercial option [17,18,19,20].

To enhance the strengths of the two polymers and optimize their cost and benefits, several studies have investigated blends of PLA and TPS. However, it is important to consider that TPS is hydrophilic, while PLA is hydrophobic, resulting in high tension at the interface between both biopolymers, poor adhesion, and lack of compatibility [21,22,23]. Therefore, improving the adhesion interface between the two components is crucial for an effective PLA/TPS blend. The scientific literature suggests various alternatives, such as incorporating coupling agents like maleic anhydride into the PLA, which contain functional groups capable of interacting with the hydroxyl groups present in TPS [23,24,25]. Another widely used alternative is incorporating glycerol, a plasticizer that enhances the thermoplasticity of TPS and improves compatibility with other polymers [22,26]. Furthermore, the compatibilization of PLA/TPS using glycidyl methacrylate (GMA) has recently been explored, resulting in compatibilized blends with superior mechanical properties compared to non-compatibilized ones [27,28,29,30]. Considering the importance of chemical modification to enhance the interaction between PLA and TPS, this paper reports the functionalization of commercial maize starch with GMA, plasticized with glycerol (Gly) or garlic oil (GO), for use as a compatibilizer in PLA/TPS blends. It is important to mention that the use of GO serves two purposes: 1) to evaluate its plasticizing capability in comparison to Gly, and 2) to explore the antioxidant properties conferred to the film. After the production of these compatibilizers, they were blended with PLA and TPS. Dicumyl peroxide (DCP), a peroxide, was used to promote the grafting of the compatibilizer onto PLA. The effect of compatibilization was investigated in terms of structural, morphological, thermal, mechanical, and antioxidant properties.

## 2. Materials and Methods

### 2.1. Reagents

PLA (Luminy^®^ LX175) was provided by Totalenergies (The Netherlands) and TPS (Unik Bropar PG1017) was kindly provided by Sacos 88—Sociedade de Plásticos, Lda company (Portugal). Glycerol (Gly), dicumyl peroxide 98% (DCP), and glycidyl methacrylate 99% (GMA) were purchased from Alfa Aesar (Germany), while garlic oil “Saluten” (GO) was purchased from Essencia d’um Segredo (Portugal), and commercial starch was obtained from a commercial market (maize starch, average composition of 75% amylopectin and 25% amylose). Distilled water was used for the contact angle (CA) tests. For the antioxidant activity, absolute methanol and radical 2,2-diphenyl-1-picrylhydrazyl (DPPH) were acquired from Merck (Darmstadt, Germany).

### 2.2. Film Preparation

Pristine PLA was dried at 80 ⁰C overnight in a vacuum oven. Initially, several compositions were performed using a Haake Rheometer batch mixer (Haake Rheomix Roller Rotors R600, volume 69 cm^3^) with counter-rotating rotors to determine the most feasible functionalized starch with GMA and Gly (StF) percentage for PLA/TPS matrix incorporation. Two StF percentages were tested (5 and 10 wt.%). The 10 wt.% value showed good compatibilization according to electron microscopy analysis (see Figure S1 in Supplementary Materials). Therefore, the StF content to be added was fixed at 10 wt.% of the total PLA/TPS weight (a ratio of 90/10 PLA/TPS blend was used). For the development of the compatibilizers StF and StFGO (functionalized starch with GMA and GO), the components were initially mixed in a plastic bag for previous manual mixing. This mixture was processed in a Haake batch mixer at 160 °C and 80 rpm for 15 min.

The compositions PLA/TPS (control), PLA/TPS/StF, PLA/TPS/StFGO, PLA/TPS/StF_1.5GO, and PLA/TPS/StF_3GO were also blended in a Haake batch mixer at 160 °C and 80 rpm. The components were added in the following order: first PLA and then after 1 min, TPS and the compatibilizer (StF or StFGO). At 2.5 min, DCP was introduced, and the mixture was allowed to react for 7.5 min. In the case of the compositions with GO, it was added 1 min after the DCP addition.

The chemical description of the compatibilizers and the prepared compositions are depicted in Table 1 and Table 2, respectively.

**Figure 1:**
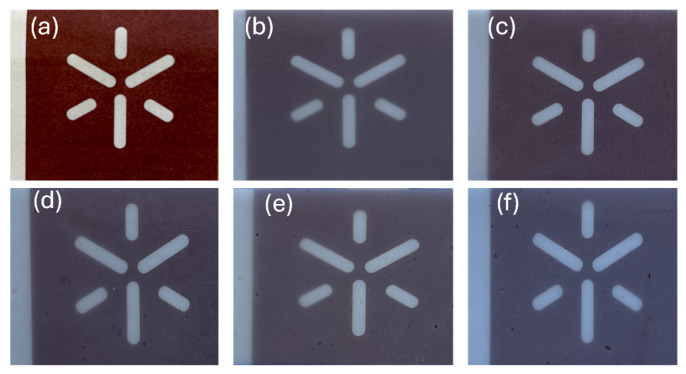
(**a**) Control; (**b**) PLA/TPS; (**c**) PLA/TPS/StF; (**d**) PLA/TPS/St/GO; (**e**) PLA/TPS/StF/1.5GO; (**f**) PLA/TPS/StF/3GO.

The compression molding technique was used to produce the composition films. Typically, this technique involves two cycles: one for heating, during which the material is melted and compressed, followed by a cooling cycle to stabilize and consolidate the resulting film. Approximately 5 g of each composition was melted at 170 °C for 5 min, and then a pressure of 20 tons was applied for 3 min. Cooling was achieved in the press using water circulation. The photographs of the resulting films are shown in Figure 1, where it is possible to observe that the incorporation of compatibilizers does not affect the transparency of the films.

### 2.3. Films Thickness

The film thickness of the different films was measured using a digital micrometer gauge (Mitutoyo Absolute, Japan). The presented values are the average of at least 5 random readings on each film sample.

### 2.4. Structural and Morphological Characterization

Fourier transform infrared spectroscopy (FTIR) analysis was performed in a Perkin Elmer (Waltham, MA, USA) Spectrum 100 spectrometer in ATR mode with 16 accumulations, 4 cm^−1^ resolution, and a range of 4000–700 cm^−1^.

Morphological evaluation was achieved in an ultra-high-resolution field emission gun scanning electron microscopy (FEG-SEM, Hillsboro, OR, USA), NOVA 200 Nano SEM (FEI Company, Hillsboro, OR, USA). Samples were previously fractured in liquid nitrogen and covered with a thin film (2 nm) of Au-Pd (80–20 weight %) in a high-resolution sputter coater (208HR Cressington Company, Watford, UK), coupled to an MTM-20 Cressington High-Resolution Thickness Controller.

### 2.5. Thermal Characterization

Thermogravimetric analysis (TGA) of the films was carried out in a TGA Q500 (TA Instruments, New Castle, DE, USA) under a nitrogen atmosphere at 10 °C/min in a temperature range from 40 to 500 °C.

A DSC Netzsch 200 Maya (Netzsch, Selb, Germany) was used to access the melting and crystallization temperature of the crystalline phase, under a nitrogen atmosphere and a heating rate of 10 °C/min. Two heating cycles were performed, the first to erase the thermal history of the samples and the second to analyze the data. The crystallinity degree ($X_{c}$) was determined using Equation (1) [31]:

(1) $$X_{c}(\%)=\frac{∆H_{m}}{∆H_{m}^{0}\times f}\times 100$$

where $∆H_{m}$ is the experimental enthalpy of fusion of the sample, $∆H_{m}^{0}$ is the theoretical heat of fusion for 100% crystalline PLA (93.7 J/g [32]), and f is the weight fraction of PLA.

### 2.6. Mechanical Characterization

Tensile tests were conducted using a Zwick/Roell Z005 universal testing machine (Zwick/Roell, Ulm, Germany), in accordance with the ASTM D882–02 standard. To ensure accuracy, at least 5 specimens (25 × 150 mm) were employed, with a grip separation of 100 mm and a crosshead velocity of 5 mm/min. This analysis was performed to determine the Young’s modulus (E), strain ($\varepsilon_{br}$) and stress at break ($\sigma_{br}$).

### 2.7. Contact Angle (CA)

The contact angle (CA) was used to estimate the surface hydrophobicity of the films. It was measured using a goniometer (Contact Angle System OCA 20 Dataphysics, Germany). The CA measurement with water was conducted on a flat film surface and analyzed using the acquired images. A precise syringe was used to drop 3 µL of distilled water onto the film surfaces following the sessile drop method. The initial image of the drop (captured at 0 s) was recorded using a video camera. To obtain the mean value, a minimum of 20 measurements per film were performed.

### 2.8. Antioxidant Activity

The DPPH radical assay is a simple and fast method to evaluate the antioxidant activity of a given sample. Briefly, a methanolic DPPH solution (2 mL, 14.2 μg/mL) was added to 50 μL of the sample. The solutions were homogenized and kept in darkness for 30 min. The absorbance was measured at 515 nm. The inhibition percentage was measured using Equation (2).

(2) $$IP(\%)=\frac{AC-AS}{AC}\times 100$$

Here, AC stands for the control’s absorbance and AS stands for the sample’s absorbance. The applied approach was according to Andrade et al.’s method [33].

### 2.9. Statistical Analysis

The results of each test represent the mean values and standard deviation of three samples, analyzed by analysis of variance (ANOVA) and post hoc Tukey test, using the SPSS v.22.0 program (IBM Corp., Armonk, NY, USA).

## 3. Results and Discussion

### 3.1. Structural and Morphological Characterization

#### 3.1.1. Fourier Transform Infrared Spectroscopy (FTIR)

**Figure 2:**
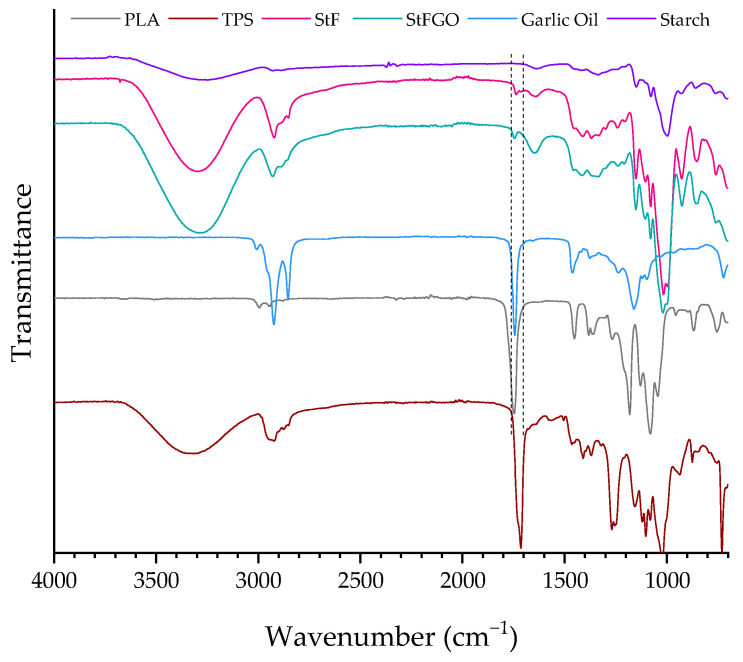
FTIR spectra of the raw materials and the functionalized starches.

**Figure 3:**
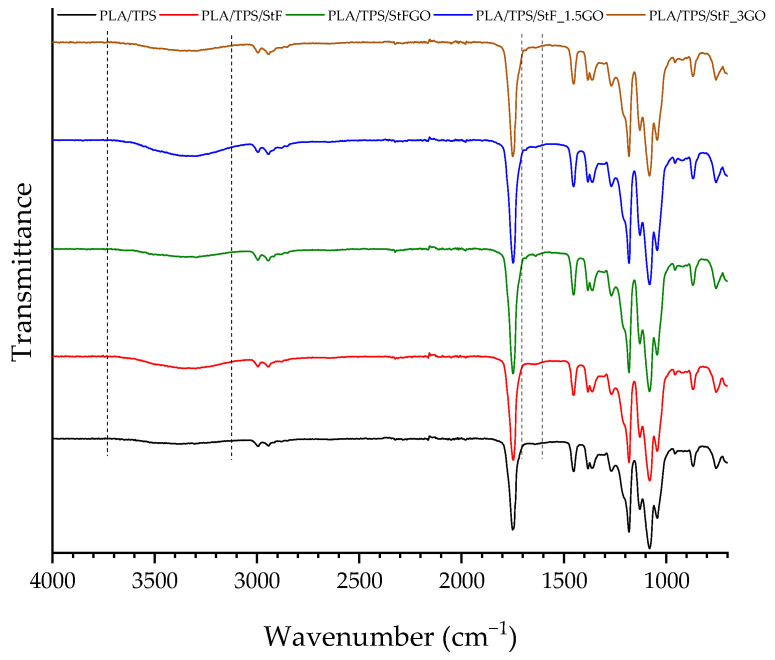
FTIR spectra of the produced compositions.

To verify any structural modifications due to chemical reactions during processing, all samples were analyzed using FTIR. The spectra of the compatibilizer and the prepared compositions are shown in Figure 2 and Figure 3, respectively. Table 3 summarizes the characteristic band’s wavenumber of each material. The spectrum corresponding to PLA shows a peak at approximately 1743 cm^−1^ corresponding to the stretching of the C=O bond, due to the presence of carbonyl groups in the PLA structure. Between 1382 and 1352 cm^−1^ and 2943 and 3000 cm^−1^, the bands are related to the symmetric and asymmetric vibration of the -CH group from the hydrocarbon chain of the polymer. Another characteristic peak is located at approximately 1182 cm^−1^ and corresponds to the asymmetric stretching vibrations of the C-O-C bond, which is typical of the PLA chain [23,34]. TPS spectrum reveals several characteristic peaks (Figure 2 and Table 3), as already observed in the literature [35]. A broad band around 3325 cm^−1^ reflects the presence of hydroxyl groups (O-H), characteristic of starch. Another characteristic peak is found between 2874 and 2951 cm^−1^, corresponding to the stretching vibrations of the C-H bonds. At approximately 1700 cm^−1^, another peak is detected, which corresponds to the carbonyl group (C=O) of the polyester. According to the material’s technical data sheet, this is an additive in its composition. Additionally, peaks at 1031 cm^−1^ and 1160 cm^−1^ are related to C-O-C asymmetric stretching and C-O stretching, respectively. Garlic contains many biological components, including organosulfur compounds, polyphenols, polysaccharides, vitamins, and proteins [36]. Since the used GO is a commercial oil, its detailed chemical composition is unknown; however, some specific bands can be identified. The bands at 2850 and 2980 cm^−1^ are assigned to CH symmetric and anti-symmetric stretching, and the band at 1710 cm^−1^ is attributed to the carbonyl group (C=O) of proteins. The band at 1400 cm^−1^ corresponds to the stretching of the methyl group, primarily from lipids, and the vibration of the diallyl sulfide molecule is observed at 1192 cm^−1^. Regarding the neat starch, characteristic peaks include a broad peak from 3590 to 3040 cm^−1^, corresponding to the O–H stretching of amylopectin; a peak at 2920 cm^−1^, associated with C-H stretching; a peak at 1645 cm^−1^, related to C-O bending (associated with the OH group); peaks from 1390 to 1465 cm^−1^ related to C-H bending; and a peak at approximately 1000 cm^−1^ related to ether groups (C-O-C). For the compatibilizers developed in this study (StF and StGO), the spectra are very similar to that of neat starch, with no significant differences observed except for the presence of new peaks around 1734 cm^−1^ and 1750 cm^−1^ for StF and StGO, respectively, corresponding to the stretching of the carbonyl bond (C=O) present in GMA and GO (in the case of StGO). This new peak indicates the successful functionalization of starch with GMA.

Regarding the FTIR spectra of the blends containing PLA, TPS, and functionalized starches, Figure 3 and Table 3 show no significant differences between the control composition PLA/TPS spectrum and the other compositions. The characteristic peaks from the PLA and TPS polymers, previously described, overlap with possible changes in the matrix, particularly in the regions of carbonyl (C=O) and ether (C-O-C) group stretching. However, a slight shoulder is noticeable between 1640 and 1688 cm^−1^, resulting from the incorporation of the starches StF and StFGO. Peaks that could confirm the reaction of PLA and GMA appear around 1180 cm^−1^ and 1017 cm^−1^, indicating the C-O stretching in the ester and hydroxyl groups, respectively. However, these peaks overlap with the characteristic peaks of PLA and TPS [30].

#### 3.1.2. Scanning Electron Microscopy

**Figure 4:**
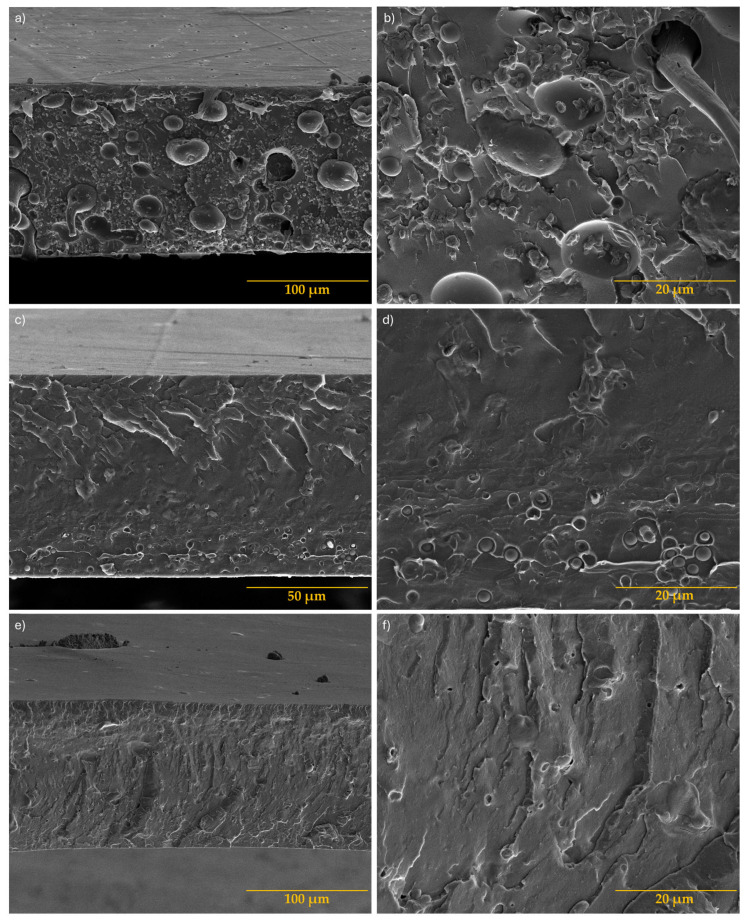

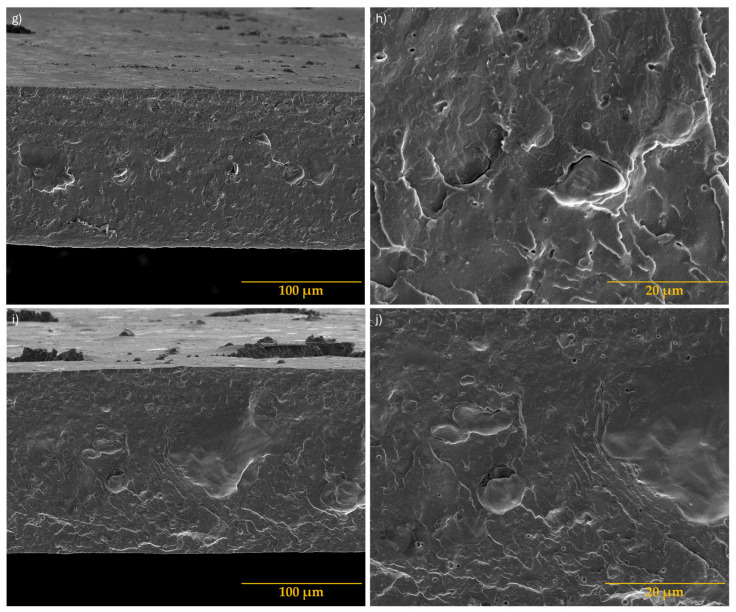
SEM images of PLA/TPS (**a**,**b**); PLA/TPS/StF (**c**,**d**); PLA/TPS/StFGO (**e**,**f**); PLA/TPS/StF_1.5GO (**g**,**h**); and PLA/TPS/StF_3GO (**i**,**j**). Images (**a**,**c**,**e**,**g**,**i**) are in 100 µm scale, and (**b**,**d**,**f**,**h**,**j**) are in 50 µm scale.

Morphological evaluation was performed to assess the homogeneity of the compositions and investigate the interfacial adhesion between the components. The magnified morphologies at 1000× and 5000× are shown on the left and right sides of Figure 4, respectively.

Analyses of Figure 4a,b reveal that the fracture of the PLA/TPS film exhibits an uneven topography with distinct phases. The images show dispersed spherical particles of TPS (dispersed phase) with low interaction with the PLA matrix (continuous phase), attributed to a lack of adhesion between the two polymers, as previously mentioned [24]. The incorporation of functionalized starches significantly increases compatibilization between PLA and TPS. Comparing both functionalized starches (StF with GMA and Gly; and StFGO with GMA and GO), it can be seen that the latter appears more effective in improving compatibilization and interfacial adhesion, as shown in Figure 4c–f. The compositions of PLA/TPS/StF_1.5GO and PLA/TPS/StF_3GO in Figure 4g–j exhibit a considerably more homogeneous and continuous morphology than the previous compositions, indicating an excellent interface between both phases. Moreover, an increase in GO content leads to better interfacial adhesion among all components. It is also important to note that the presence of DCP and GMA in all the produced blends positively affects the morphology, making it difficult to distinguish between the two phases in most blends, which aligns with initial expectations. The inclusion of peroxide induces the formation of free radicals that play a fundamental role in the chemical reaction between the functionalized starches and the PLA chain. This means that the peroxide creates radicals along the hydrocarbon chain of PLA, which subsequently reacts with the double bond in the GMA agent present in StF and StFGO. Thus, promoting covalent bond grafting improves the dispersibility of the TPS granules in the PLA matrix and reinforces the interfacial bonding between PLA and TPS [29,30].

### 3.2. Thermal Characterization

**Figure 5:**
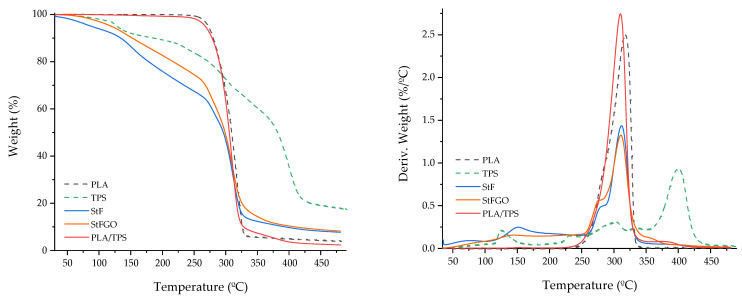
TGA thermograms of the neat PLA and TPS, the compatibilizers StF and StFGO, and the control blend PLA/TPS.

**Figure 6:**
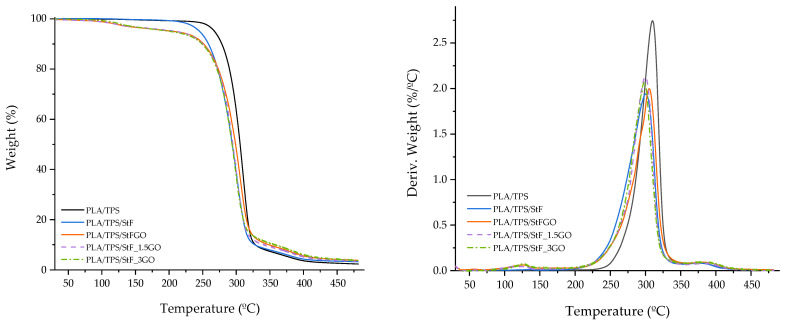
TGA thermograms of the control blend PLA/TPS and the produced compositions.

**Figure 7:**
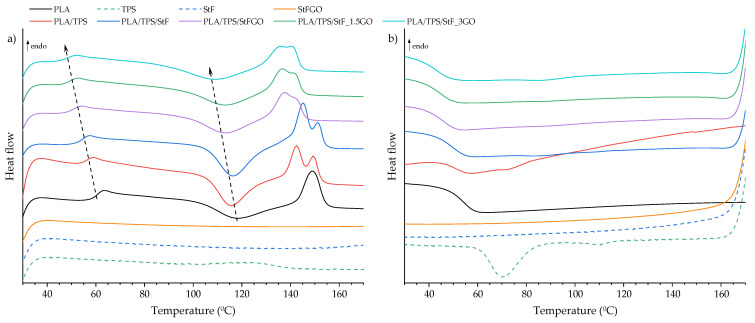
DSC thermograms of the neat polymers, the compatibilizers, and the compositions produced during (**a**) the second heating and (**b**) the first cooling cycle.

To investigate the influence of the developed compatibilizers on the thermal properties of the produced systems, TGA and DSC thermograms were performed on both the functionalized starches and compositions, as shown in Figure 5, Figure 6 and Figure 7. More detailed data are presented in Table 4, where T_peak_, Tg, T_c_, T_m_, ΔH_m_, and X_c_ correspond to the degradation peak temperature, glass transition temperature, cold crystallization temperature, melting temperature, melting enthalpy, and the degree of crystallinity, respectively. These curves represent the first cooling and second heating of the samples to eliminate the thermal history of the polymers.

From Figure 5 and Table 4, it can be observed that the addition of TPS does not significantly impact the thermal stability of the matrix. The degradation temperature decreases from 317 °C to 310 °C, and the Tg value decreases from 59 °C to 54 °C. However, Xc significantly increases from 22 to 31 %, indicating improved reorganization of the PLA molecules. Comparing the TPS with the StF and StFGO thermograms, three degradation stages can be seen, indicating similar degradation patterns. TPS alone presents a crystallization peak of its crystalline phase during cooling, with a corresponding melting peak at 123 °C (no cold crystallization is observed), consistent with previously reported values [37]. Nevertheless, it is evident that the developed compatibilizers exhibit lower thermal stability. Additionally, the thermal stability of StF is slightly lower than that of StFGO.

Concerning the analyses of the PLA/TPS/StF(GO) systems, the TGA thermograms indicate good thermostability of these materials (Figure 6). From Table 4, it can be observed that the main degradation peak temperatures range from 298 to 310 °C with a weight loss between 84 and 91 %. The Tg values decrease (from 59 °C to 45 °C) with the incorporation of the compatibilizer and increasing GO content, indicating enhanced interaction between PLA and TPS. As reported by Esmaeili et al., the plasticizers present in TPS may migrate and interact with the PLA phase, increasing chain mobility [38]. Additionally, the presence of GO affects the organization of the crystalline phase. This is evidenced by the decrease in the cold crystallization peak (indicating that molecules can rotate and organize at lower temperatures, as indicated by Tg) and melting temperature (the peak decreases with a broader transformation, suggesting that the crystalline phase is less organized). This can be explained by the increased compatibility resulting from greater intermolecular interactions between TPS and PLA, which hinders the organization of the PLA crystalline structure. Similar findings have been reported in the literature [27]. Regarding the crystallinity degree, it generally increases compared to PLA; however, this varies depending on the composition. Specifically, PLA/TPS/StF has the highest (37%), while PLA/TPS/StF_1.5GO has the lowest (25%). Additionally, the presence of TPS disrupts PLA crystallinity, as observed by the appearance of two distinct melting peaks (Figure 7 and Table 4). In the analysis of the cooling cycle (Figure 7b), crystallization is not visible, suggesting that the cooling rate was high enough to inhibit crystal growth. Therefore, the crystalline structure forms during heating, as indicated by the presence of cold crystallization peaks (T_c_). The T_c_ values between 100 and 120 °C are often attributed to the formation of a mixture of two crystalline polymorphs, δ and α forms (T_m_ of 137 and 142 °C, respectively) [39,40]. However, it is important to evaluate the spatial conformation of the crystals using XRD to identify the type of crystalline phase.

### 3.3. Mechanical Characterization

**Figure 8:**
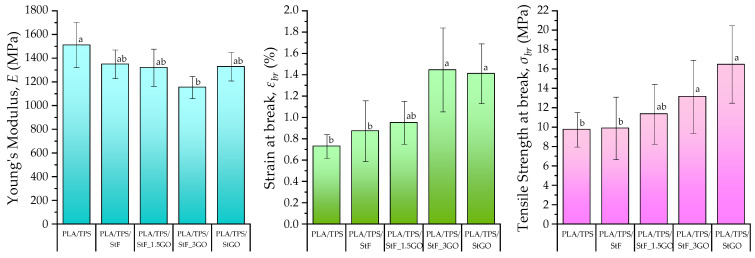
Mechanical properties (Young’s modulus (E), strain ($\varepsilon_{br}$) and tensile strength ($\sigma_{br}$) at break) for the produced blends (samples with arithmetic means that do not share a letter are significantly different).

Figure 8 depicts the mechanical indexes for each PLA/TPS blend obtained through tensile strength–strain tests (tensile strength–strain curves are present in Figure S2). Due to the lower Young’s Modulus of TPS compared to PLA, the blend reduces the value from 3500 MPa (from the PLA technical datasheet) to 1500 MPa. The incorporation of a compatibilizer is expected to decrease the interfacial energy between the incompatible phases (PLA and TPS), resulting in improved energy transfer and enhanced interfacial adhesion. Additionally, the particle size of the dispersed phase (TPS) decreases, as observed in the SEM analysis. These effects are evident in the increased deformation ability and strength of the blends, as the formation of crazes and microcracks is delayed [41]. This influence is more pronounced in the blends compatibilized with GO, showing improvements of 93 and 68% in $\varepsilon_{br}$ and $\sigma_{br}$, respectively, for the PLA/TPS/StGO blend. These results are consistent with the results of morphological analysis. Garlic oil alone has a more positive influence than glycerol, as it interacts better with the OH groups in PLA/TPS, forming stronger hydrogen bonds among all the components.

### 3.4. Contact Angle

**Figure 9:**
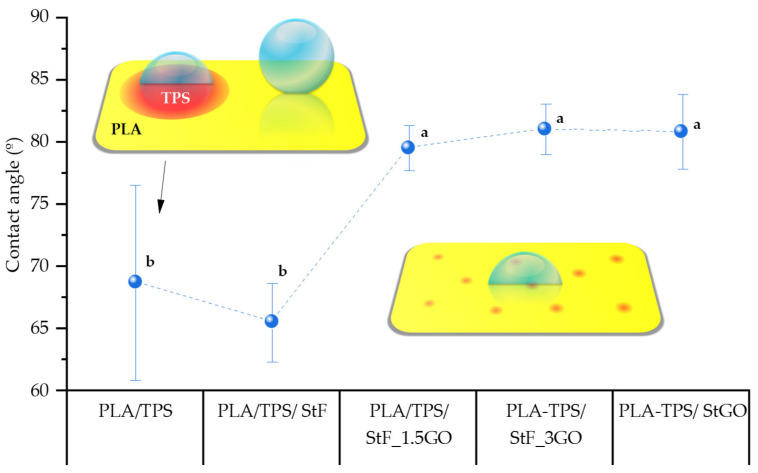
Contact angles of the produced films (samples with arithmetic means that do not share a letter are significantly different).

The analysis of contact angles is crucial for understanding the hydrophilicity or wettability of the film. This phenomenon relates to the film material’s ability to adsorb liquid, with higher contact angles indicating lower wettability. Since the film surfaces were not entirely homogeneous, contact angle measurements were taken at several different points. Figure 9 presents illustrations of droplets dispersed on the films taken immediately after dispensing, along with the average contact angle values for each film. As can be seen, the PLA/TPS blend exhibits the highest standard deviation, suggesting that the TPS phase is poorly dispersed in the matrix. Areas with a higher concentration of TPS show a lower contact angle due to their hydrophilic nature. The figure indicates that the contact angle decreases from 69° to 65° with the incorporation of functionalized starch with GMA and Gly into the PLA/TPS matrix. Rosa Turco et al. also observed this effect with the addition of a plasticizer [42]. Thus, this result may indicate that the hydrogen bonds between StF and the matrix orient the polar groups of the functionalized starch toward the surface, increasing the film’s hydrophilicity. In contrast, incorporating GO into the PLA/TPS systems leads to an increase in the contact angles of the compositions, specifically, 80° for StFGO, 79° for PLA/TPS/StF_1.5GO, and 81° for PLA/TPS/StF_3GO. However, despite these findings, all film surfaces exhibit hydrophilic properties, indicating that the films’ chemical composition shapes their hydrophobic characteristics.

### 3.5. Antioxidant Activity

Antioxidant activity plays a crucial role in food packaging because exposure to oxygen can accelerate food deterioration, reducing its shelf life. To determine if processing affected the biological properties of garlic oil, the DPPH test was conducted to assess the antioxidant activity of the compositions. UV-Vis analysis of the solutions was performed at the time of the test and again after 48 h to evaluate the films’ performance in terms of biological activity. The results are presented in Table 5.

Based on the activity values obtained, the control solution containing only GO exhibits the highest activity in both analyses (0 and 48 h). Among the compositions with GO, PLA/TPS/StFGO shows the highest values, with 18.1% and 42.4% DPPH scavenging activity, indicating a greater capacity to neutralize free radicals compared to the other compositions and therefore better antioxidant activity. This is followed by PLA/TPS/St_3GO with 17.3% and 36.4%, and then PLA/TPS/St_1.5GO with 11.1% and 20.3%, at 0 h and 48 h, respectively. This result was expected because the first composition has the highest content of GO and the antioxidant activity of garlic is well known and supported by previous studies. For example, Teixeira et al. [12] investigated the antioxidant capacity of garlic essential oil in fish protein films, while Rojas et al. [43] observed that low concentrations of active agents, such as garlic, resulted in significant inhibition of microbial growth and strong antioxidant activity in active PLA films.

Surprisingly, the compositions PLA/TPS and PLA/TPS/StF do not contain any active agents, yet they still exhibit antioxidant activity. This can be attributed to the presence of additives incorporated into neat polymers, which may contain polyphenols, compounds with antioxidant properties.

It is worth noting that all the compositions tested show a significant increase in DPPH scavenging activity after 48 h, indicating that the films have a controlled release of the active agent (GO), an important characteristic for films used in the food packaging industry.

## 4. Conclusions

This study demonstrated that the incorporation of two compatibilizers derived from commercial starch, functionalized with GMA and plasticized with GLY or GO, significantly improved the miscibility of PLA/TPS systems. This enhancement was attributed to better interfacial adhesion between the components. Although the thermal stability of the PLA/TPS/StF(GO) system decreased slightly compared to pure PLA, it remained within acceptable limits, indicating good thermal performance.

Crystallinity improved with the addition of compatibilizers, particularly with higher GO content, though the crystalline phase was less stable, as evidenced by a lower melting temperature compared to neat PLA. Mechanical properties followed a similar trend, with GO incorporation leading to enhanced medium resistance, making the films mechanically stronger than unmodified PLA/TPS systems.

The wettability of the films also improved substantially with increasing GO content, a feature that can be particularly beneficial for applications requiring better printability and sealing capabilities. Additionally, GO concentration significantly influenced the films’ antioxidant activity, with higher GO levels enhancing the scavenging of DPPH radicals. This was further supported by the methodology’s ability to enable a controlled release of GO active agents, providing sustained antioxidant activity over time.

In summary, the developed methodology for producing PLA/TPS/StF(GO) systems not only improves compatibility, thermal, mechanical, and wettability properties but also introduces a biodegradable material with antioxidant properties. These features make the system a promising candidate for biodegradable packaging films, particularly for applications requiring extended shelf life and enhanced functional performance.

## Figures and Tables

**Table 1:** Composition of the StF and StF_GO compatibilizers.

Compositions	St (Maize) (wt.%)	GMA (wt.%)	Gly (wt.%)	GO (wt.%)
StF	55	10	35	
StF_GO	55	10		35

**Table 2:** Composition and thickness of the produced films.

Composition	PLA (wt.%)	TPS (wt.%)	StF (wt.%)	StFGO (wt.%)	GO (wt.%)	DCP (wt.% of SfF)	e_film_ (mm)
PLA/TPS	90	10					0.08 ± 0.01
PLA/TPS/StF	81	9	10			1	0.11 ± 0.04
PLA/TPS/StFGO	81	9		10		1	0.13 ± 0.01
PLA/TPS/StF_1.5GO	79.6	8.9	10		1.5	1	0.12 ± 0.01
PLA/TPS/StF_3GO	78.3	8.7	10		3	1	0.15 ± 0.02

Note: e_film_ is the final average thickness of the films obtained by compression molding.

**Table 3:** Characteristic bands wavenumber of neat PLA and TPS and modified systems.

Composition	Wavenumber (cm^−1^)	Bond
PLA	1182	C-O-C
	1352–1382; 2943–3000	C-H
	1743	C=O
TPS	3340	O-H
	2874–2951	C-H
	1031, 1160	C-O
Starch	3590–3040	O-H
	2920, 1465–1390	C-H
	1645	C-O
	1000	C-O-C
StF	1734	C=O
StFGO	1750	C=O
PLA/TPS/StF	1688; 1640	C=O
PLA/TPS/StGO	1688; 1640	C=O
PLA/TPS/StF_1.5GO	1688; 1640	C=O
PLA/TPS/StF_3GO	1688; 1640	C=O

**Table 4:** Thermal parameters obtained from the TGA and DSC analyses.

Composition	T_peak_ (°C)	T_g_ (°C)	T_c_ (°C)	T_m_ (°C)	$∆H_{m}$ (J/g)	$X_{c}\;(\%)$
PLA	317	59	118	149	20.7	22
TPS	125; 302; 396	–	70	123	3.9	–
StF	150; 312	–	–	–	–	–
StFGO	140: 310	–	–	–	–	–
PLA/TPS	310; 382	54	116	142; 150	25.8	31
PLA/TPS/StF	300; 382	51	116	145; 153	27.7	37
PLA/TPS/StGO	126; 305; 388	47	113	138;142	20.9	27
PLA/TPS/StF_1.5GO	121; 300; 386	46	112	137;142	18.7	25
PLA/TPS/StF_3GO	127; 298; 387	45	107	136;140	23.2	32

Note: Tpeak is the degradation temperature at maximum DTG peak; Tg is the glass transition temperature; Tc is the cold crystallization temperature, and Tm is the melting temperature, $∆H_{m}$ is the melting enthalpy, and $X_{c}$ is the degree of crystallinity.

**Table 5:** Antioxidant activity of the compositions produced, measured with the free radical scavenging method.

Compositions	DPPH Scavenging Activity (%)	
	0 h	48 h
PLA/TPS	10.5	28.8
PLA/TPS/St	15.8	37.1
PLA-TPS/St/GO	18.1	42.4
PLA/TPS/St_1.5GO	11.1	20.3
PLA/TPS/St_3GO	17.3	36.4
GO	22.2	71.2

## Data Availability

The data presented in this study are available in the article.

## Supplementary Materials

The following supporting information can be downloaded at: www.mdpi.com/article/10.3390/antiox13121589/s1.
