# Supplementary material for: A New Approach in PLS/TPS Compatibilization Using Garlic Oil: Effect on Morphological and Antioxidant Properties
Source: Antioxidants (Basel). 2024 Dec 23;13(12):1589. doi: 10.3390/antiox13121589 (PMC11672901; doi:10.3390/antiox13121589)
Supplement: Supplementary file 1 [file antioxidants-13-01589-s001.zip › antioxidants-3236131-supplementary.pdf]

Supplementary material

A New Approach in PLS/TPS Compatibilization Using Garlic Oil: Effect on Morphological and Antioxidant Properties

Supplementary materials:

Figure S1 illustrates the morphology of the TPS phase dispersed within the PLA matrix. In the PLA/TPS blend, it is evident that there is minimal interaction between the TPS phase and the PLA matrix. However, the addition of 5% StF compatibilizer significantly reduces the interfacial tension between the two polymers, with this effect becoming more pronounced at a 10% compatibilizer concentration.

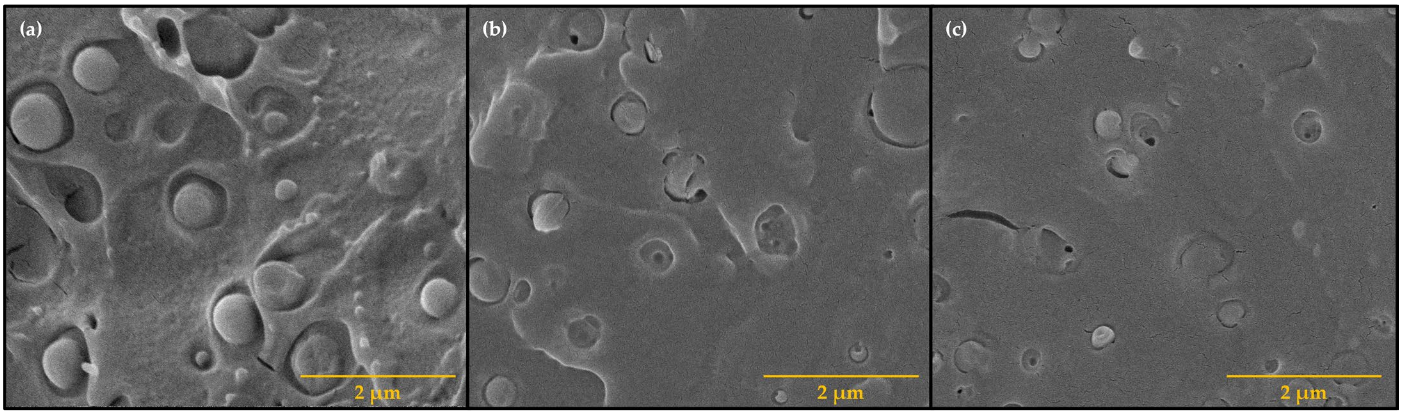

Figure S1. SEM images of (**a**) uncompatibilized PLA/TPS and (**b**,**c**) compatibilized PLA/TPS blends with 5% and 10% StF.

Figure S2 illustrates the tensile-strain curves of the produced blends: PLA/TPS, PLA/TPS/StF, PLA/TPS/StF_1.5GO, PLA/TPS/StF_3GO and PLA/TPS/StFGO.

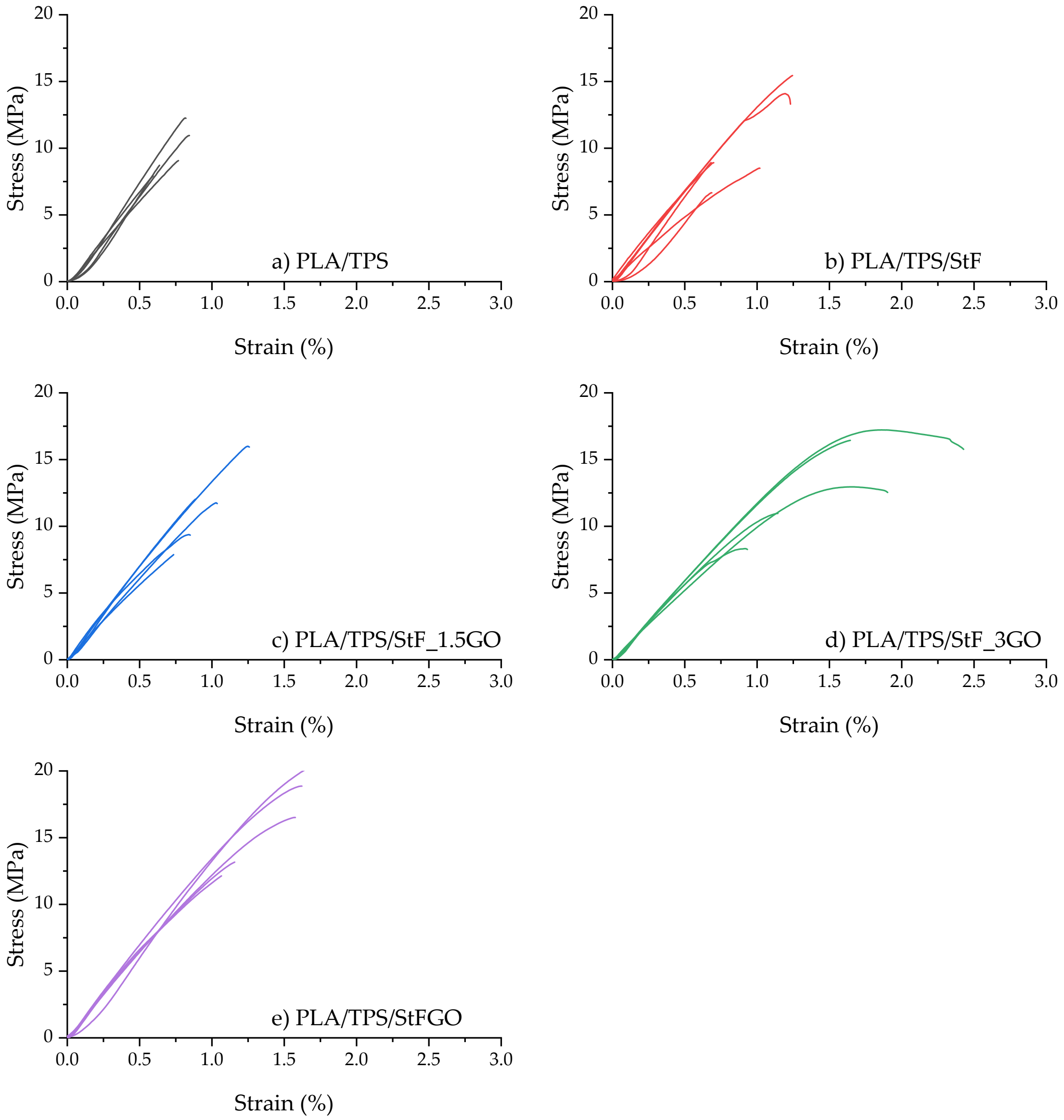

Figure S2. Tensile-stress curves of PLA/TPS blends: (**a**) PLA/TPS, (**b**) PLA/TPS/StF, (**c**) PLA/TPS/StF_1.5GO, (**d**) PLA/TPS/StF_3GO and (**e**) PLA/TPS/StFGO.
